# Gut microbial signatures in poultry under low-protein diets and lysine restriction: implications for precision nutrition

**DOI:** 10.1016/j.vas.2026.100848

**Published:** 2026-08-31

**Authors:** Ahmad Salahi, Wafaa A. Abd El-Ghany

**Affiliations:** a Department of Animal Science, Faculty of Agriculture, University of Zanjan, Zanjan, Iran; b Department of Poultry Diseases, Faculty of Veterinary Medicine, Cairo University, Giza, Egypt

**Keywords:** Poultry, Gut microbiome, Low-protein diet, Lysine restriction, Microbial signatures, Precision nutrition, SCFAs

## Abstract

Three key signatures — microbial, nutritional, and epigenetic — are pivotal in nutrigenomics, mediating poultry responses to nutritional signals, such as low-protein diets (LPDs) and lysine restriction (LR). These multidimensional signatures — encompassing bacterial, archaeal, and fungal taxonomy, functional genes, and metabolomic profiles (short-chain fatty acids; SCFAs) — orchestrate host metabolism, immunity, and feed conversion ratio (FCR). This PRISMA-ScR scoping review synthesizes multi-omics evidence showing that LPDs shift cecal fermentation toward saccharolytic pathways, enriching butyrate-producing taxa (*Ruminococcaceae, Lachnospiraceae*) while suppressing Proteobacteria and toxic proteolytic metabolites (ammonia, branched-chain fatty acids). LR elevates *Actinobacteria*/*Synergistetes*, triggers microbial nitrogen limitation, and reconfigures SCFA profiles through cross-feeding, thereby activating host nutrient sensors (mTOR downregulation, GCN2 upregulation) to prioritize immunity and FCR gains. Methanogenic archaea (*Methanobrevibacter*) fine-tune hydrogen economies, although diet-induced changes require deeper exploration. Key signatures forecast performance, featuring optimized Firmicutes/Bacteroidetes ratios and ureolytic genes (*gdhA*/*glnA*) as markers of protein resilience and profiles for breed-specific responses. SCFAs drive epigenetics (HDAC suppression, histone acetylation) and immunometabolism (Treg expansion, NF-κB dampening), bridging microbiome to host benefits via gut-liver interplay. Emerging applications span next-generation probiotics (NGPs) and live biotherapeutic products (LBPs), tailored from signature consortia (L. *reuteri* + B. *velezensis*) or archaeal agents, to sustain diet-induced states in antibiotic-free regimens. Major gaps persist in causal multi-omics validation, breed-specific responses, standardized baselines, and archaeal contributions. Integrating LPD/LR with tailored NGPs/LBPs promises precision nutrition strategies that enhance nitrogen utilization, reduce emissions, and improve welfare. Gnotobiotic, FMT, and AI trials are essential for translating microbial signatures into scalable flock-level precision management.

## Highlights

• Low crude protein (LCP) diets and lysine restriction reshape poultry gut microbial signatures toward saccharolytic taxa and SCFA production.
• Microbial signatures (*Lactobacillus* enrichment, reduced *Proteobacteria*) serve as biomarkers for feed efficiency under protein restriction.
• Indole-derived AhR signaling represents a potential mechanism linking diet-induced microbiota shifts to immunity and performance.
• Precision nutrition integrating microbiome signatures with multi-omics enables flock-specific protein optimization.
• Next-generation probiotics targeting signature consortia offer sustainable alternatives to antibiotics in low-protein regimens.

## Graphical abstract

**Figure:**
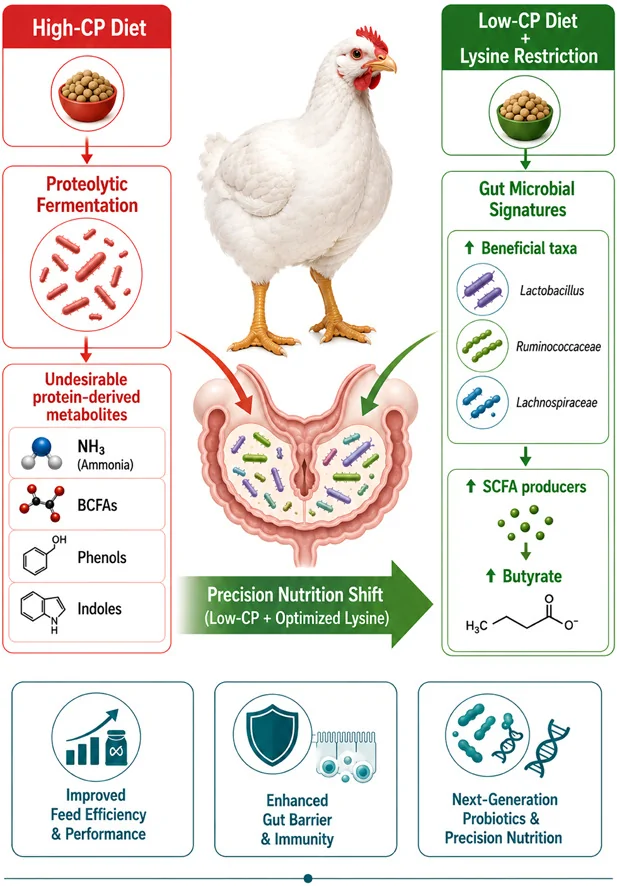

## 1 Introduction

The gut microbiome comprises bacteria, archaea, fungi, viruses, and protozoa that collectively function as an active metabolic and endocrine partner of the host. The avian gut microbiome exhibits compartment-specific patterns along the gastrointestinal tract—spanning twelve distinct segments from the beak to the cloaca—where diverse microorganisms collectively orchestrate a unified symphony of metabolic and physiological functions. In chickens and turkeys, analysis of high-quality 16S rRNA sequences revealed 915 and 464 species-equivalent OTUs, respectively, spanning 13 phyla with Firmicutes, Bacteroidetes, and Proteobacteria dominating (>90% of sequences), with core genera including Clostridium, *Ruminococcus, Lactobacillus*, and *Bacteroides* (Wei et al., 2013). On commercial European broiler farms, the intestinal microbiota exhibits a highly compartmentalized structure, where the ileal microbiota is overwhelmingly dominated by *Lactobacillus* spp. (∼83%), followed by *Escherichiacoli*(∼4%), *Enterococcus* spp. (∼3%), members of the family Lachnospiraceae (∼2%), and a diverse assemblage of other taxa (∼8%). In contrast, the cecal microbiota is enriched in obligate anaerobes, with *Lachnospiraceae* (∼47%) and Ruminococcaceae (∼19%) as the predominant families, alongside *Bifidobacterium* (∼10%), *Lactobacillus* (∼10%), *Coriobacteriaceae* (∼7%), *Bacteroides* (∼2%), and other minor taxa (∼5%), reflecting the specialized fermentative function of the ceca (Apajalahti & Vienola, 2016).

In healthy humans, the gut microbiome comprises approximately 10¹⁴ bacterial cells, with species diversity exceeding 1000 species (∼1150 in Europeans (Qin et al., 2010); 632 in Dutch (Zhernakova et al., 2016); 1235 in Chinese, (Yang et al., 2020)). In contrast, microbial density in the gastrointestinal tract of a healthy chicken increases progressively along the avian gut, ranging from approximately 10³–10⁴ CFU/g in the crop and gizzard—dominated primarily by lactobacilli and streptococci—to 10⁸–10⁹ CFU/g in the ileum, and reaching 10¹¹–10¹² CFU/g in the ceca, where *ruminococci, Bacteroides, clostridia, streptococci*, enterococci, lactobacilli, and *E. coli* collectively form a dense, metabolically active anaerobic ecosystem (Yadav & Jha, 2019). The cecal microbiota is dominated by representatives of the order Clostridiales, which account for >50% of the community, while the remainder is mainly composed of the orders Bacteroidales and Lactobacillales (Apajalahti and Kettunen, 2006; Torok et al., 2011; Zhu et al., 2002). However, unlike humans, where comprehensive milestones such as Milestones in Human Microbiota Research have been published, no equivalent large-scale, standardized datasets exist for poultry, and precise information on their microbial diversity—including alpha-diversity (the richness of microbial types within a sample) and beta-diversity (the difference in microbial composition between samples) —remains limited. Additionally, while a definitive milestone revised the human intestinal surface area from ∼200 m² to 30–40 m² (Helander & Fändriks, 2014), an equivalent pivotal study recalibrating this metric in poultry has not yet been published.

The gut microbiome serves as an active endocrine partner, secreting small proteins and other molecules that influence host physiology and immunity in humans (Duan et al., 2024; Sberro et al., 2019) and affect the quality of poultry meat and eggs. In avian species, these small, largely uncharacterized proteins and molecules highlight the potential for microbial-informed precision nutrition strategies. This review synthesizes evidence on diet-responsive microbial signatures in poultry, focusing on low-protein (LP) diets and lysine restriction (LR), their effects on microbiota composition, metabolism, and performance, and their potential utility as biomarkers for precision nutrition and next-generation probiotics (NGPs). From a nutrigenomics perspective, diet-induced signatures—such as metabolic (e.g., SCFA production and nutrient-sensing pathways), epigenetic (e.g., HDAC inhibition and histone modifications), and microbial profiles (e.g., taxonomic shifts and functional gene enrichment)—have been extensively investigated in humans for personalized nutrition. In contrast, these signatures, particularly those responsive to low-protein diets and lysine restriction, remain underexplored in poultry, representing a substantial opportunity to develop precision nutrition strategies.

### 1.1 Microbial signatures as the language of precision nutrition

Microbial signatures—multidimensional profiles integrating taxonomy (bacteria/archaea/fungi/viruses), functional capacity, metabolomics (SCFAs), and archaeal H₂ metabolism—characterize host-microbiome interfaces more robustly than diversity metrics alone (Krummenauer et al., 2025; Oke et al., 2025; Schwendner et al., 2022). Future microbial signature frameworks should further incorporate cross-kingdom interactions among bacteria, archaea, fungi, viruses, and protozoa, rather than focusing predominantly on bacterial communities alone (Zhang et al., 2026). In poultry, the gut microbiota function as bioreactors, transforming dietary substrates into epigenetic modulators (histone acetylation/DNA methylation) along the gut-liver axis, shaping metabolism, nutrient acquisition, stress responses, and disease resistance (Hoye & Fenton, 2018; Naeem & Fatima, 2025; Sang et al., 2025). Notably, SCFAs derived from microbial fermentation shape the gut physicochemical environment by lowering luminal pH and reducing oxygen availability, thereby constraining the expansion of aerotolerant pathogens and stabilizing functional microbial signatures (Steinert et al., 2025). Emerging evidence indicates that SCFAs and polyamines may influence bacterial membrane potential (Vmem), potentially contributing to gut–brain signaling (Lombardo-Hernández et al., 2025). Unlike alpha (Shannon) or beta (Bray-Curtis) diversity, these functional fingerprints yield actionable precision nutrition biomarkers (Berg & Cernava, 2022; Gunjur et al., 2024; Liu et al., 2022). Recent poultry multi-omics studies further demonstrate that integrating cecal microbiome profiles with host metabolomics reveals microbial–metabolic signatures associated with growth performance, highlighting the value of functional rather than purely taxonomic biomarkers (Zhao et al., 2026). In humans, microbial signatures are highly individualized and can serve as a novel forensic tool alongside traditional methods like fingerprints and DNA, enabling the identification of individuals through unique microbial traces left on surfaces during interactions—such as in mock crime scenes—while skin microbiomes remain relatively stable in composition and taxonomic proportions absent major disturbances, with even identical twins exhibiting distinguishable profiles (Hampton-Marcell et al., 2020). This individuality and forensic applicability underscore the potential for microbial signatures to serve as discriminative biomarkers in poultry, where breed-specific responses to dietary interventions, such as LPD/LR, further highlight their predictive utility. Longitudinal analysis in mice revealed significant time-dependent shifts in gut microbial β-diversity under both low-protein (3%) and adequate-protein (12%) diets, highlighting dynamic community restructuring during protein modulation (Masuoka et al., 2020).

Poultry exemplifies microbiome-driven precision nutrition: rapid growth, lysine sensitivity, feed efficiency, and dependence on intestinal function enable reproducible diet-microbiome-phenotype links (Hu et al., 2025). Controlled mouse models confirm that low-protein feeding (3% crude protein) alters both host nutritional status and gut microbiota composition relative to protein-adequate diets (12% crude protein) (Masuoka et al., 2020). Protein/lysine restriction restructures communities toward performance-linked signatures (Wu et al., 2022; Xiong et al., 2024), offering scalable alternatives to FCR prediction. Breed-specific colonization (Arbor Acres vs. Tibetan) underscores predictive power (Hu et al., 2025). Notably, in mice fed a 3% protein diet, body weight loss occurred despite unchanged food intake, indicating protein-specific effects rather than energy-driven mechanisms (Masuoka et al., 2020).

NGPs/LBPs target diet-induced signatures to achieve antibiotic-free metabolic resilience (Hua et al., 2025; Kujawska & Hall, 2025; MTIG & Barberio, 2024; Teigen, 2025). Accordingly, this PRISMA-ScR scoping review synthesizes multi-omics evidence on diet-responsive microbial signatures in poultry fed low-protein diets (LPDs) or subjected to lysine restriction (LR), elucidating their roles in microbiota restructuring, host metabolism, immunity, and feed efficiency, as well as their potential as biomarkers for precision nutrition and next-generation probiotics/live biotherapeutic products (NGPs/LBPs).

### 1.2 Research gap and future directions: microbial signatures induced by protein reduction and lysine restriction

Critical gaps persist in defining functional microbial signatures that integrate taxonomic composition, SCFA/metabolic capacity, and amino acid-linked pathways (i.e., metabolon data) in poultry fed low-protein (LP) and lysine-restricted (LR) diets. Despite mechanistic insights from mouse models manipulating dietary protein (3%vs. 12%), the precise links between gut microbiota dynamics and host nitrogen balance remain incompletely resolved and largely unexplored in poultry systems (Masuoka et al., 2020). Current evidence, predominantly correlational (16S rRNA/PICRUSt-dominant), documents taxonomic/SCFA shifts linked to performance but lacks causal depth from gnotobiotic/FMT models, limiting mechanistic insight (Forcina et al., 2022). Moreover, substantial microbial dark matter—including uncultured microorganisms and underexplored non-bacterial kingdoms (e.g., viruses and protozoa)—remains unresolved in poultry, limiting comprehensive microbial signature discovery and functional annotation (Zhang et al., 2026). Although recent poultry studies have begun integrating microbiomics with metabolomics to identify microbial–metabolic signatures associated with growth traits, comparable multi-omics investigations under low-protein and lysine-restricted nutritional strategies remain scarce (Zhao et al., 2026).

Crucially understudied, LP formulations inevitably elevate starch content, thereby confounding protein digestion and amino acid utilization. 75–80% of amino acids are absorbed via PepT-1 as di- or tripeptides (Gilbert et al., 2008; Salahi et al., 2025); reduced reliance on intact protein and crystalline amino acids depletes luminal peptides while glucose competition intensifies (Macelline et al., 2021). Rapidly digestible starches (wheat> corn/sorghum) and excessive starch: protein ratios drive hyperglycemia, hyperinsulinemia, abdominal fat, and impaired performance (Chrystal et al., 2020; Liu et al., 2021). Imbalanced rations may increase gut permeability, intestinal dysbiosis, and harmful bacteria, impairing digestive function and accelerating chronic liver disorders (Salahi et al., 2025b). The starch–glucose–insulin–microbiome axis remains systematically unexplored under LP/LR regimens (Table 1).

**Table 1:** Identified research gaps in microbial signature studies on low-protein and lysine-restricted diets.

Domain	Knowledge Gap	Key Question	Proposed Approach	Reference
Predictive Validation	No framework for lysine-induced signatures as FCR biomarkers	Can AAs turnover patterns predict precision nutrition responses?	1. Shotgun metagenomics+metabolomics 2. ML modeling FCR correlation	(Gunjur et al., 2024; Schwendner et al., 2022)
Causal Evidence	No causal link diet→signatures→host epigenetics/immunity	Do SCFAs causally drive mTOR/GCN2 immune outcomes?	1. Gnotobiotic+FMT 2. Multi-tissue omics 3. Engineered strains	(Naeem & Fatima, 2025; Sang et al., 2025)
Breed Effects	Unknown breed-signature plasticity under LR	Can AA signatures transfer to Tibetan chickens?	1. FMT trials AA→Tibetan 2. Multi-omics tracking 3. FCR validation.	(Hu et al., 2025)
NGP/LBP Pipeline	No validated translation signature→regulatory LBP	Can consortia mimic LR signatures safely?	1. Signature isolation 2. Gnotobiotic safety 3. LBP assays	(Hua et al., 2025; MTIG & Barberio, 2024)
Methodological Standardization	Non-standardized protocols → irreproducible signatures	How to create comparable poultry signatures?	1. Contamination controls 2. Shotgun+avian databases 3. Open pipelines	(Bautista et al., 2025)
Microbial Mechanisms	Unknown lysine transporters/metabolic flux in poultry bacteria	How bacteria sense/process lysine → signature?	1. Isolate+genome seq 2. ¹³C-lysine flux 3. In vitro omics	(Schwendner et al., 2022)
Temporal Dynamics	Unknown signature stability/timeline post-diet	Transient vs. stable LR signatures?	1. Daily sampling 2. Network analysis 3. Diet re-challenge	(Hu et al., 2025)
Nutrient Interactions	Starch confounding in LP microbiome effects	Starch: protein ratio → signatures?	Starch-controlled multi-omics trials	(Gilbert et al., 2008)

**Abbreviations:** AAs: Amino acids, AA: Arbor Acres, FCR: Feed Conversion Ratio, FMT: Fecal Microbiota Transplantation, GCN2: General Control Nonderepressible 2, LBP: Live Biotherapeutic Product, mTOR: mechanistic Target of Rapamycin, NGP: Next-Generation Probiotic, SCFAs: Short-Chain Fatty Acids.

Lysine-graded modulation of microbial outputs, mTOR/GCN2 signaling, and breed-specific responses lack standardized multi-omics integration (shotgun metagenomics, metabolomics, and transcriptomics). Recent evidence suggests that individual essential amino acids exert distinct effects on gut microbial composition and amino acid transport under low-protein diets, emphasizing the need to distinguish lysine-specific microbial signatures from broader amino acid balance effects (Liang et al., 2026). Longitudinal baselines are absent, hampering the development of precision biomarkers. Thus, resilience may be explained by extensive microbial micronutrient sharing, whereby gut bacteria form metabolically cooperative networks that exchange vitamins, cofactors, and bioenergetic molecules to stabilize community function under dietary constraints (Steinert et al., 2025).

Future priorities include causal longitudinal trials, gnotobiotic/FMT validation, AI-driven predictive modeling, starch-standardized protocols, and archaea-targeted NGPs/LBPs. On-farm qPCR biomarkers, breed-optimized thresholds, and regulatory LBP frameworks will transform LP/LR strategies from economic tactics into sustainable precision nutrition paradigms—redefining gut health, microbial function, and resilient poultry systems (Taylor-Bowden et al., 2024). Achieving these goals will require standardized, interoperable microbiome reference databases that support AI-driven analyses, improve cross-study comparability, and accelerate robust microbial signature discovery (Zhang et al., 2026).

## 2 Methods: scoping review protocol (PRISMA-ScR)

This scoping review followed PRISMA-ScR guidelines for systematic evidence mapping, key concept identification, and clarification of knowledge gaps regarding gut microbial signatures in poultry fed low-protein (LP) and lysine-restricted (LR) diets. A comprehensive literature search covered PubMed, Scopus, Web of Science, and Google Scholar for publications from January 2015 to February 2026 (updated as of 2026– 02– 07, including preprints and early-view articles to capture emerging poultry omics data). Boolean combinations targeted host species, dietary interventions, microbiome signatures, and host-microbe mechanisms; example PubMed string: (poultry OR broiler OR layer) AND ("low protein" OR "protein restriction" OR "lysine restriction") AND ("gut microbiome" OR "microbial signature" OR microbiota OR archaea) AND (metabolomic OR SCFA OR mTOR OR GCN2 OR immune). Relevant review reference lists were manually screened for supplementary studies.

**Fig. 1:**
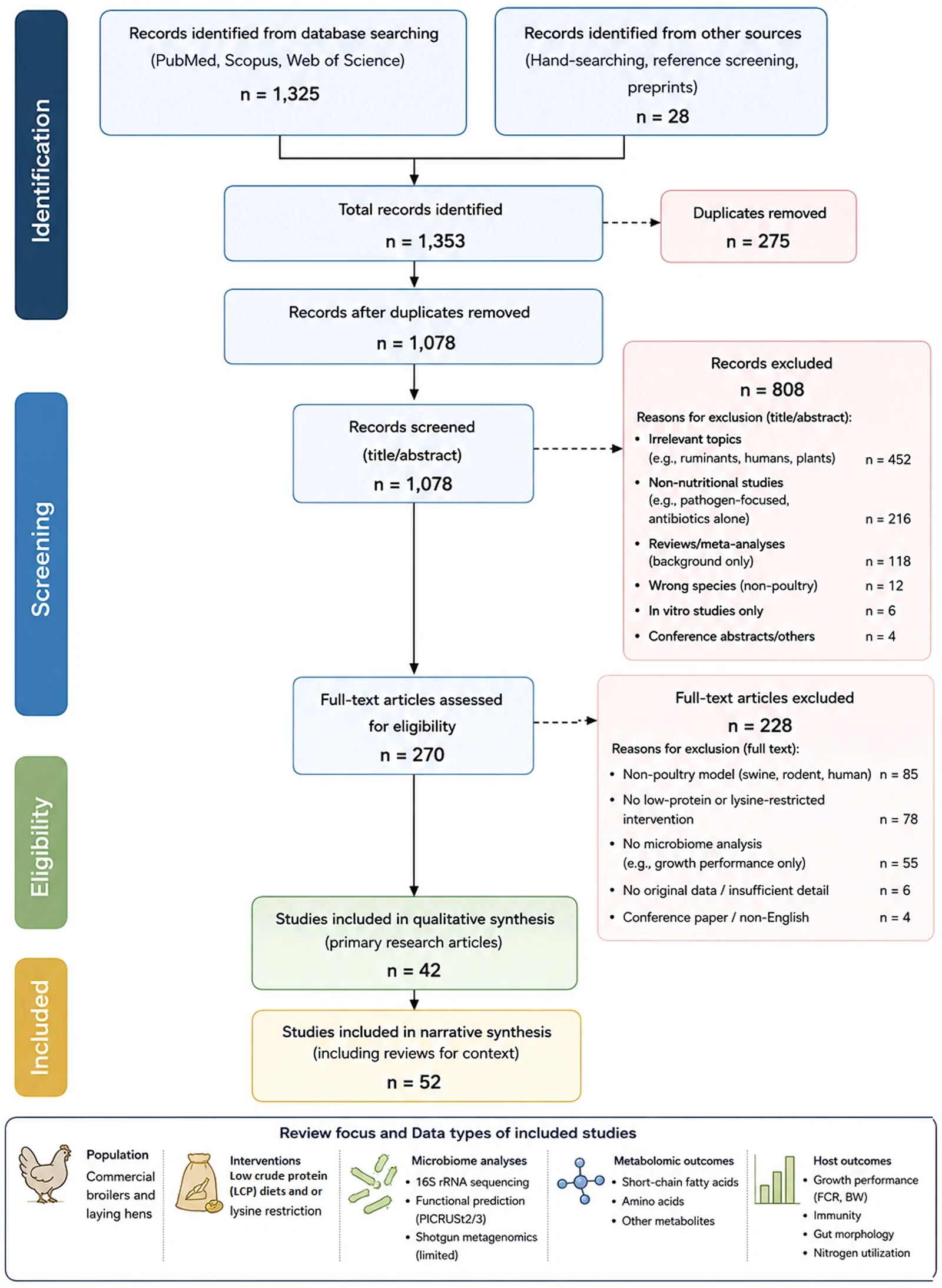
PRISMA-ScR flow diagram illustrating the study selection process for the scoping review of gut microbiome signatures in poultry under low-protein diets and lysine restriction.

Studies underwent dual-reviewer screening (kappa = 0.87 inter-rater reliability) at the title/abstract and full-text stages, with discrepancies resolved by consensus of the senior author. Predefined eligibility criteria required: Population—commercial broilers or laying hens; Intervention—LP or LR diets versus adequate controls; Outcomes—gut microbial signatures encompassing (1) taxonomic shifts (16S rRNA sequencing), (2) functional potential or activity (mainly PICRUSt2/3 predictions from 16S data; shotgun metagenomics/metatranscriptomics limited to <10% of studies), and (3) metabolite profiles (SCFAs/amino acids via metabolomics). Designs included primary in vivo experiments, observational cohorts, and pertinent systematic reviews. Exclusions comprised pathogen-only, in vitro fermentation, or non-English studies. The process yielded 1325 identified records (plus 28 from other sources), 1078 after deduplication (275 duplicates removed), 1078 screened at the title/abstract, 270 full-text assessed, 228 full-text excluded, and 42 studies included in qualitative synthesis, and 52 studies included for narrative synthesis (Fig. 1).

Data extraction used standardized templates to capture study attributes (author/year/country/breed/age), interventions (crude protein/digestible lysine levels/duration), methods (16S region/sequencing platform/metabolomics), and outcomes (alpha/beta-diversity, phylum/genus shifts, SCFAs, host phenotype correlations). This mechanistic emphasis enables computational modeling of dietary effects by adjusting metabolite fluxes or through microbial knockouts (Jansma & El Aidy, 2021). Marked heterogeneity across designs/diets/methods precluded quantitative meta-analysis. Narrative synthesis used vote-counting to assess directional trends (e.g., 80% of studies (16/20) reported increased SCFAs/butyrate under LP, with qualitatively moderate to large effect sizes per available forest plots).

## 3 Baseline gut microbiome and signature profiles in poultry

The avian gastrointestinal tract harbors a complex, compartmentalized microbial ecosystem essential to poultry health and productivity. The ileum and jejunum maintain low microbial diversity dominated by lactic acid bacteria, with *Lactobacillus* spp. comprising>90% of the population alongside *Enterococcus* and *Streptococcus* (Apajalahti & Vienola, 2016; Rychlik et al., 2020). Studies confirm that up to 95% of small-intestinal bacteria produce lactic acid, with phyla including Actinobacteria, Proteobacteria, and others (Apajalahti & Vienola, 2016; Taylor-Bowden et al., 2024). Conversely, the cecum serves as the primary fermentation chamber, hosting dense communities (∼10¹⁰ CFU/g digesta) and ∼1000 species (Apajalahti & Vienola, 2016; Rychlik et al., 2020). *Firmicutes* and *Bacteroidetes* dominate the ceca of broilers/layers (83–92%), driving carbohydrate fermentation and SCFA production (Ban et al., 2025; Oke et al., 2025). Cecal density exceeds that of the ileum by 100–1000-fold, and diet principally shapes composition, making the cecum a bioreactor for health- and productivity-related metabolites (Apajalahti & Vienola, 2016; Naeem & Fatima, 2025; Oke et al., 2025).

Health-linked ecosystem-level configurations correlate with growth, FCR, and physiology through optimal Firmicutes/Bacteroidetes (F/B) ratios that support nutrient efficiency, barrier integrity, and immunity (Jansma & El Aidy, 2021; Taylor-Bowden et al., 2024). Importantly, reduced dietary protein does not necessarily disrupt dominant phyla, as Firmicutes and Bacteroidetes remain prevalent under low-protein regimens in finishing pigs, supporting the concept of a resilient core microbiome under protein restriction (Wang et al., 2023). SCFA producers (*Ruminococcaceae: Faecalibacterium, Ruminococcus, Butyricicoccus, Butyricimonas*) generate butyrate via acetyl-CoA/succinate pathways (Liu et al., 2023; Rychlik et al., 2020); *Bacteroides* and *Colidextribacter* regulate immunity; *Barnesiella* and *Alistipes* boost SCFAs (Liu et al., 2023). Arbor Acres (AA) chickens enrich growth taxa (*Erysipelatoclostridium, Hydrogenoanalobacterium, Shuttleworthia*); Tibetan breeds favor immune taxa (*Limosilactobacillus, Ligilactobacillus, Prevotella*) (Hu et al., 2025). Dysbiosis features depleted *Firmicutes*/*Bacteroidetes*, expanded *Proteobacteria*/*Enterobacteriaceae*, inflammation, and poor FCR from excess protein proteolysis (ammonia, biogenic amines, BCFAs) (Apajalahti & Vienola, 2016; Elling-Staats et al., 2022; Liu et al., 2023; Shazali et al., 2019).

**Fig. 2:**
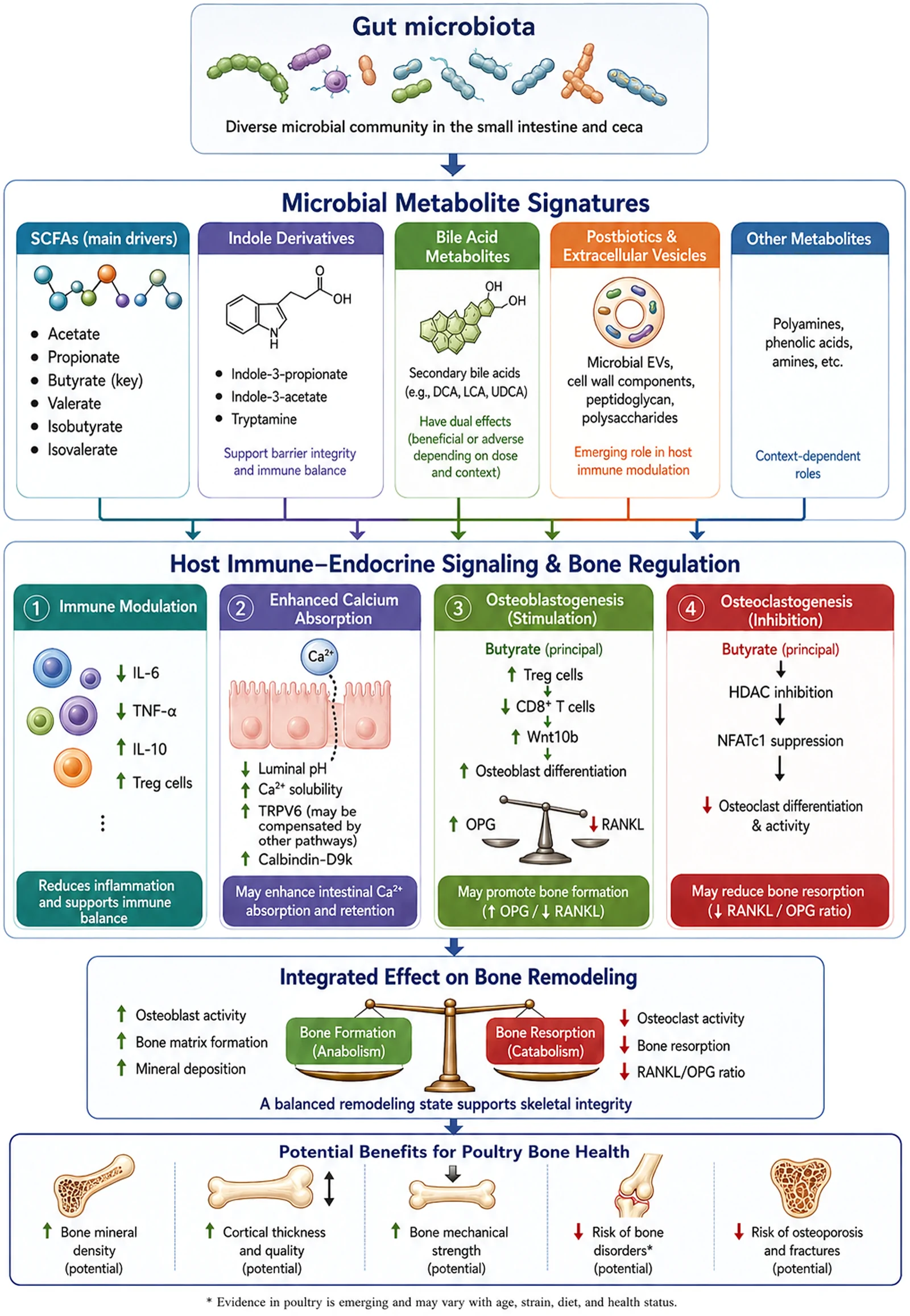
The gut-bone axis in poultry: microbiota-SCFA-bone interactions.

Archaea (∼1%) exert metabolic influence: *Methanobrevibacter* consumes H₂ via methanogenesis, with syntrophic interactions, CAZyme activity, and SCFA production benefits (Deng et al., 2021; Elling-Staats et al., 2022; Salahi et al., 2025). Interventions include reducing protein intake, supplementing with amino acids, and prebiotics (inulin, fructo-oligosaccharides) and xylanase to sustain beneficial signatures (Apajalahti & Vienola, 2016; Elling-Staats et al., 2022; Shazali et al., 2019). Critically, no standardized "healthy baseline signature" exists, nor are lysine-responsive taxa defined—core gaps for precision nutrition (Taylor-Bowden et al., 2024). Beyond metabolic and immune outcomes, SCFA-driven microbial signatures also intersect with the gut–bone axis (Fig. 2), influencing mineral absorption and skeletal integrity.

Beyond bacteria and archaea, the avian gut harbors a minor but highly dynamic fungal community (mycobiota) that exhibits pronounced spatial heterogeneity and striking inter-study variability (Yadav & Jha, 2019). Unlike bacterial communities, which increase in density and diversity toward the ceca, the chicken intestinal mycobiota is consistently more diverse in the upper gastrointestinal tract (crop, proventriculus, duodenum, jejunum, ileum) and markedly reduced in the ceca and colon (Robinson et al., 2022). The mycobiota is dominated by *Ascomycota* and *Basidiomycota*, with *Gibberella* (*Fusarium pseudonygamai*), Candida, and Aspergillus as recurrent genera across studies. However, >50% of fungal ASVs are study-specific, reflecting strong sensitivity to the housing environment and, most critically, dietary fungal inputs. Notably, dietary sources rapidly replace hatchery-derived fungi within three days post-hatch, establishing feed as the primary determinant of mycobiota structure (Robinson et al., 2022). These findings indicate that, although quantitatively minor (<0.06% of bacterial load), the intestinal mycobiota represents a highly plastic ecological layer that may modulate gut physiology and metabolite exposure, warranting inclusion in future multi-kingdom microbial signature frameworks. Given the strong dietary dependence of fungal colonization, protein source and feed processing may indirectly shape exposure to mycobiota-derived metabolites under low-protein feeding strategies, introducing an additional, largely unexplored layer of diet–microbiome interaction in poultry.

## 4 Microbial signatures induced by low-protein diets

**Fig. 3:**
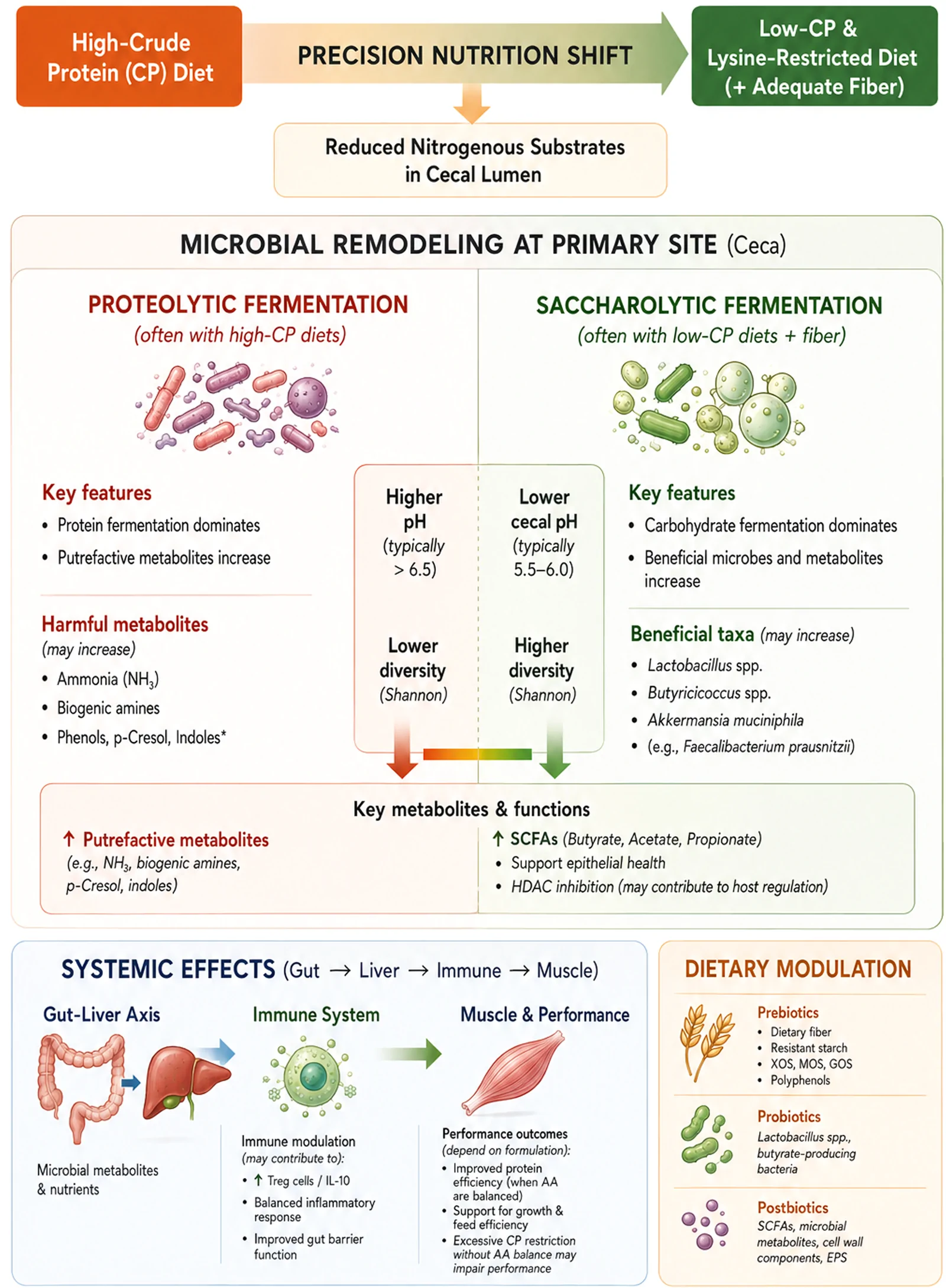
Microbial and metabolic reprogramming in broilers: the shifts to low-CP & lysine-restricted precision nutrition.

Low-protein diets (LPDs) supplemented with essential amino acids redirect microbial fermentation from proteolytic (protein) to saccharolytic (carbohydrate) pathways, markedly suppressing toxic protein-fermentation metabolites (ammonia, biogenic amines, phenols, BCFAs) (Apajalahti & Vienola, 2016; Elling-Staats et al., 2022). This saccharolytic reprogramming appears conserved across monogastric species, as reduced dietary protein similarly shifts the gut microbiota toward carbohydrate-fermenting taxa in finishing pigs (Wang et al., 2023). Mechanistically, recent mouse studies demonstrate that low-protein diets remodel white adipose tissue through microbiota-dependent bile acid–FXR signaling in adipose progenitors and nrfA-expressing bacteria-derived ammonia inducing hepatic FGF21. In this microbiota-dependent response, *Romboutsia timonensis* and *Bilophila* spp. were identified as key microbial mediators that enhance bile acid signaling and hepatic FGF21 induction, thereby promoting beige adipocyte differentiation and increased energy expenditure (Tanoue et al., 2026). However, whether a similar mechanism exists in poultry remains unknown because classical brown and beige adipose tissues have not been clearly identified in birds. This results in a decline in cecal pH, which inhibits protein breakdown (Elling-Staats et al., 2022). Host-preferred nutrient absorption exacerbates microbial nitrogen limitation—a dynamic amplified under LPDs (Reese et al., 2018). In mice, severe protein restriction (3% crude protein) selectively enriched urease-producing bacterial taxa, indicating microbial adaptation to nitrogen scarcity via alternative nitrogen acquisition strategies (Masuoka et al., 2020). Conversely, carbohydrate fermentation surges, yielding beneficial SCFAs (butyrate, acetate, propionate) via anaerobic cecal breakdown (Liu et al., 2023; Mátis et al., 2013). SCFA production decreases luminal pH and reduces oxygen availability, thereby limiting the overgrowth of aerotolerant and opportunistic pathogens and reinforcing ecosystem-level colonization resistance (Steinert et al., 2025). It may also contribute to bioelectrical signaling in the gut–brain axis (Lombardo-Hernández et al., 2025). Genera such as *Butyricimonas* and *Ruminococcus* directly convert sugars to butyrate (Liu et al., 2021), bolstered by cross-feeding (*Eubacterium hallii*, Ana*e*rostipes coli) via butyryl CoA: acetate CoA transferase—a conserved mechanism across species (Louis et al., 2022; Muñoz-Tamayo et al., 2011). Butyrate and propionate act as HDAC inhibitors, driving histone hyperacetylation with epigenetic potency (Mátis et al., 2013, Wang et al., 2025). Saccharolytic shifts also modulate amino acid metabolism, yielding nitric oxide, polyamines, and indole derivatives that govern intestinal and systemic immunity (Oke et al., 2025) (Fig. 3).

Characteristic community-level fingerprints feature elevated Shannon diversity, enriched lactic/butyrate producers (*lactobacilli, Ruminococcaceae, Lachnospiraceae; Faecalibacterium prausnitzii, Roseburia*) (Hou et al., 2020), immune regulators (*Bacteroides, Colidextribacter*), and depleted pathogens (Proteobacteria/Enterobacteriaceae, Cyanobacteria) (Liu et al., 2021; Shazali et al., 2019). Conversely, mice fed protein-adequate diets (12% crude protein) exhibited increased abundance of *Streptococcaceae* and *Lachnospiraceae*, reflecting distinct microbial metabolic strategies under sufficient nitrogen availability (Masuoka et al., 2020). Consistent with poultry findings, low-protein diets increased the abundance of *Lactobacillus* and butyrate-associated genera in pigs, reinforcing the role of LPDs in selecting health-promoting, fermentation-efficient microbial consortia (Wang et al., 2023). Evidence from monogastric models further supports this concept, as low-protein diets increased ileal and cecal microbial richness and diversity in finishing pigs, whereas excessive protein intake reduced microbiome diversity and impaired gut stability (Wang et al., 2023). Dynamic cross-feeding (SCFAs/H₂/acetate) sustains stability, fueling methanogens (Horrocks et al., 2023; Patel, 2025). *Methanobrevibacter* may remove excess bowel hydrogen and thereby support microbial fermentation and energy extraction (Kim et al., 2026). Optimal dosing: 2% crude protein reduction + lysine/methionine/threonine, though imbalance risks growth/health deficits (Chen et al., 2025; Shazali et al., 2019).

These shifts yield superior gut morphology (↑villus height/crypt ratio), humoral immunity (↑IgM/IgG), barrier strength, and ↓inflammation (Liu et al., 2021; Shazali et al., 2019). SCFAs energize epithelia and modulate metabolism, immunity, and integrity (Patel, 2025); *Oscillospiraceae*/*Lachnospiraceae* limit pathogens via fiber-derived butyrate (Hao et al., 2021). Mechanistically, butyrate/propionate dose-dependently tighten junctions via HDAC–LOX activation (Mátis et al., 2013); SCFA mixtures preserve TER, ZO-1, occludin, and claudin-1 against LPS (Feng et al., 2018).

Immunomodulation: butyrate promotes Th1 but blocks Th17/IL-23/IL-17, induces Treg via Foxp3 acetylation, and boosts IL-10 (Chen et al., 2019; Furusawa et al., 2013; Zhang et al., 2016). It curbs macrophage and neutrophil pro-inflammatory responses via HDAC inhibition (context- and concentration-dependent, ↑Th1/IFN-γ at high doses) (Kespohl et al., 2017; Wang et al., 2025). Systemically, SCFAs tune distal immunity and physiology (Louis et al., 2022); nutrient shifts gate the availability of methyl donors for DNA methylation (Naeem & Fatima, 2025). NGPs (proteases/xylanases) stabilize signatures by minimizing cecal protein bypass and boosting fermentable substrates via oligosaccharides (Apajalahti & Vienola, 2016). NSP enzymes reduce viscosity, liberate nutrients, and prebiotically reshape the microbiota (Ibrahim et al., 2023). Benefits extend environmentally: ↓N excretion curbs litter ammonia via limited substrates (Anderson et al., 2020; Lambert et al., 2023), thereby orchestrating health, productivity, and resilience.

## 5 Lysine restriction–driven microbial & host signatures

Lysine, the pivotal first-limiting amino acid in poultry nutrition, underpins core metabolic and growth processes. Dietary lysine restriction (LR) markedly restructures the gut microbiome's composition and function, driving profound community shifts (Taylor-Bowden et al., 2024; Zhang et al., 2021). Even modest 20% reductions in essential amino acids are sufficient to alter beta diversity and specific bacterial abundances (Zhang et al., 2021). Phylum-level changes include enrichment of *Actinobacteria, Saccharibacteria*, and *Synergistetes*, alongside elevated family abundances (*Moraxellaceae, Halomonadaceae, Bacillaceae*) (Yin et al., 2018a). Host-preferred nutrient uptake imposes microbial nitrogen limitation, lowering fecal bacterial loads under low-protein conditions—a mechanism validated in rodents (Reese et al., 2018) and likely relevant to monogastrics like poultry. Communities rapidly exhaust simple amino acids (glycine <3% remaining within 2 weeks), signaling fierce nitrogen competition (Schwendner et al., 2022). Microbiota maturity—a key host-microbiome trait—strongly correlates with body weight (r = 0.886) and daily gain (r = 0.896), varying by genotype and amino acid supply (95.1%vs. 56.0% maturity in AA- vs. TT-restricted chickens at 14 d) (Hu et al., 2025).

Community restructuring fundamentally reshapes metabolic output, particularly SCFA profiles. Reduced microbial diversity may impair immune maturation because a diverse microbiota is essential for immune development and regulation (Kim et al., 2026). Butyrate emerges via cross-feeding: *Faecalibacterium*/*Roseburia* consume acetate, while *Eubacterium hallii* converts lactate through canonical pathways (Louis et al., 2022; Muñoz-Tamayo et al., 2011). These consortia are characteristic of high-performing chickens (Stanley et al., 2013). Notably, adequate (rather than restricted) lysine levels have been associated with elevated butyrate and suppressed branched-chain SCFAs in the cecum, highlighting complex dose-dependent effects (Konieczka et al., 2021). Beyond energetics, SCFAs serve as signaling hubs, orchestrating nutrition, energy homeostasis, community structure, HDAC/lysine deacetylase inhibition, GPCR activation, and bacterial virulence via acylation (Haque Shahid et al., 2026; Oke et al., 2025; Zhang et al., 2019). Chickens exhibit direct butyrate-driven hyperacetylation of hepatic H2A K5, cementing links to microbial epigenetics (Mátis et al., 2013). In addition to these molecular pathways, recent work indicates that diet-modulated microbial bioelectrical activity may provide an additional signaling layer, in which bacterial Vmem changes induced by SCFAs or polyamines could directly interact with enteric neurons and influence systemic neurophysiological responses (Lombardo-Hernández et al., 2025).

Concurrent host metabolic reconfiguration diverts amino acids from anabolism (mTOR suppression) toward intestinal immunity and inflammation (Bao, 2020). Piglet LR models show increased feed intake (↓leptin/CCK) and enriched microbial diversity and richness (Yin et al., 2018b)—awaiting avian confirmation given shared monogastric physiology. Imbalanced amino acids compromise immunity and gut health; excess undigested protein fuels *Clostridium perfringens* proliferation (Bao, 2020). Symbionts counter via induction of *Bifidobacterium longum*, which triggers TLR2/4–PI3K-Akt–IL-10 (Patel, 2025). Growth-phase SCFA-producers (*Phascolarctobacterium, Bifidobacterium, Lactobacillus*) expand alongside output (Sun et al., 2021); *Bacillus subtilis* 29,784 reprograms ceca toward *Ruminococcaceae/Lachnospiraceae* butyrate-producers, enabling lysine-responsive NGPs (Choi et al., 2021).

Nutrient status gates epigenetics: lysine-inclusive amino acids regulate anti-adipogenic miRNAs, shaping carcass quality (Naeem & Fatima, 2025); isoleucine boosts Sirt1 (NAD+-dependent deacetylase) in broilers (Liu et al., 2023). Antibiotics disrupt microbiomes, slashing muscle DNA methylation and SAM, DNMT3b, and METTL21C—proving causality from dysbiosis to host epigenetics (Xu et al., 2025). Critically absent: validated chains linking LR → defined signature → epigenetic shifts → phenotypes. Adaptive downregulation of SLC7A6 (L-lysine/L-arginine) during deficits underscores resource competition (Haque Shahid et al., 2026).

## 6 Archaeal signatures in response to protein and lysine restriction

Archaea, comprising ∼1% of the cecal microbiome, exert disproportionate metabolic influence in poultry (Elling-Staats et al., 2022). Methanogenic taxa (*Methanobrevibacter*) optimize fermentation efficiency by scavenging hydrogen from fermentative bacteria, thereby augmenting host energy extraction via methanogenesis within the gut's hydrogen economy (Cisek et al., 2023; Deng et al., 2021). This hydrogen sink sustains microbial homeostasis and confers indirect benefits to the host (Cisek et al., 2023). Archaeal signatures—defined as shifts in abundance, diversity, structure, or *metatranscriptomic* expression—characterize the low-diversity yet pivotal cecal consortium dominated by *Methanobrevibacter* in chickens (Salahi & Abd El-Ghany, 2025).

Direct poultry data linking low-protein diets or lysine restriction to archaeal signatures remain scarce (Jadhav et al., 2023), lagging ruminant research. No studies document lysine restriction's impact on archaeal communities or hydrogen fluxes. Knowledge gaps include: (1) ecological profiling across dietary regimens (Forcina et al., 2022; Misiukiewicz et al., 2021); (2) functional determinants of methanogenesis versus alternative sinks (acetogenesis) (Cisek et al., 2023; Misiukiewicz et al., 2021); (3) targeted modulation strategies. Cross-species evidence shows that amino acid imbalances (e.g., reduced methionine: lysine) deplete *Methanobrevibacter* (Zhang et al., 2024), yet poultry-specific metatranscriptomics—essential for capturing active metabolism beyond metagenomic taxonomy (Jovel et al., 2022)—are absent.

Low-protein/lysine restriction may perturb fermentative substrates, curtail hydrogen supply, and reconfigure cecal hydrogen economies. As an essential amino acid, lysine limitation could constrain hydrogen-producing bacteria, diminishing overall H₂ availability. In chickens—where *Lachnospiraceae*-driven acetogenesis dominates as the hydrogen sink—reduced H₂ would disrupt syntrophy, diverting fluxes and altering SCFA profiles (Cisek et al., 2023; Sergeant et al., 2014). Redirecting hydrogen sinks from methanogenesis toward acetogenesis could, in principle, improve energy harvest and reduce methane output—a hypothesis that requires direct testing in poultry. Although poultry-specific evidence remains scarce, low-protein feeding increased *Euryarchaeota* abundance in pig jejunum, suggesting that amino acid availability may influence archaeal niches across monogastric hosts (Wang et al., 2023).

Archaeal-targeted NGPs/LBPs remain speculative and require foundational causal studies. CRISPR-Cas engineering of commensals could dissect microbe-microbe dynamics and enable the development of precision biotherapeutics (Rahmati et al., 2025; Zheng et al., 2022). Archaeal responses to amino acid restriction represent an unexplored frontier; this model aims to catalyze hypothesis-driven research that bridges speculation and evidence.

## 7 The host–microbiome–metabolism and host–microbiome–immunity axes in precision nutrition

Host–gut microbiome interactions fundamentally shape physiological, immunological, and nutritional outcomes, forming a central axis for precision nutrition in poultry (Liu et al., 2023). These interactions are mediated by the microbiome's role as a dynamic metabolic bioreactor. Under dietary pressures, such as low-protein or lysine restriction, the microbiome ferments indigestible substrates into bioactive metabolites that regulate nutrient availability, host metabolism, and immune function (Jadhav et al., 2023; Krummenauer et al., 2025; Oke et al., 2025; Stüwe et al., 2025; Zeng et al., 2025).

Immunometabolism Interface

Immunometabolism explains how microbial metabolites reprogram host metabolic pathways to regulate immune cell activation, proliferation, and defense, and it has emerged as a key paradigm in livestock production (Oke et al., 2025). These metabolites enhance poultry health, disease resistance, and productivity by fortifying intestinal barrier integrity, improving energy utilization, enhancing resilience to oxidative stress, and promoting immune differentiation (Oke et al., 2025). Diet-derived bioactives often arise from host–microbial co-metabolism (Jadhav et al., 2023), and co-evolved commensals and host immunity are mutually regulated via metabolite signaling (Kim, 2018). Short-chain fatty acids (SCFAs) —alongside secondary bile acids, tryptophan-derived indoles, polyamines, and lipid derivatives—are pivotal in orchestrating innate and adaptive immunity (Campbell et al., 2023; Naeem & Fatima, 2025; Zeng et al., 2025).

Molecular Signaling Mechanisms

SCFAs (butyrate, propionate, acetate) modulate immunity through epigenetic mechanisms (HDAC inhibition, histone acetylation) and GPCR activation (GPR41/43/109A), suppressing NF-κB/MAPK pathways and cytokine expression (Kim, 2018; Naeem & Fatima, 2025; Zeng et al., 2025). In poultry, butyrate induces hepatic histone hyperacetylation, thereby bolstering barrier function and anti-inflammatory responses (Mátis et al., 2013). Dietary inputs regulate epigenetic enzymes such as Sirt1, linking nutrient sensing to chromatin remodeling (Liu et al., 2023; Sutton et al., 2024). Methyl donors (methionine, folate, betaine, choline) fuel S-adenosylmethionine synthesis, supporting DNA methylation of metabolic and immune genes (Sang et al., 2025).

SCFAs exert dose- and site-specific effects on barrier integrity via GPCRs, nuclear receptors, and epigenetic mechanisms, thereby attenuating NF-κB/MAPK (ERK) signaling (Zeng et al., 2025). Butyrate inhibits HDACs, reducing TNF-α and IL-1β and blocking IκB degradation and p65 activation in macrophages (Jiang et al., 2020; Wang & Zhang, 2025). In poultry, *Clostridium perfringens* α-toxin upregulates jejunal IL-1β, IL-6, IL-8, and TNF-α via NF-κB (Zhang et al., 2024). These effects are counteracted by microbial vesicles or l-arginine modulation (Kong et al., 2018; Zeng et al., 2024). Immune cells express GPR41/43/109A, AhR, PXR, FXR, and TGR5. Bifidobacterium longum activates PI3K-Akt/IL-10 (Patel, 2025), while tryptophan metabolites (indole, indole propionic acid) engage AhR (Jadhav et al., 2023). Butyrate reduction disrupts AMPK/PPARα signaling (Ding et al., 2024).

Nutrient-Sensing Integration

Microbial signatures intersect with host pathways such as mTOR and GCN2. Amino acid shifts activate GCN2 via uncharged tRNAs, leading to phosphorylation of eIF2α/ATF4 and triggering integrated stress responses (Grohmann et al., 2017; Masson, 2019). Salmonella infections alter AMPK/mTOR to reshape the cecal mucosa (Kogut et al., 2016); C. perfringens toxin elevates PLCγ1/AMPKα1 while suppressing mTOR (Rychlik, 2020). Glutamine drives enterocyte growth via mTOR, whereas deficiency curbs inflammation via GCN2 (Kong et al., 2018). These pathways regulate protein synthesis, autophagy, and immunity; GCN2 synergizes with inflammation to amplify innate responses (Liu et al., 2014). SCFAs induce trained immunity in myeloid cells (tolerogenic via SCFAs; context-dependent via succinate/polyamines) (Yang et al., 2025).

Breed contrasts show that fast-growing (AA chickens) enrich amino acid and energy pathways, whereas resilient breeds (Tibetan chickens) favor signal transduction and anti-inflammatory pathways (Hu et al., 2025).

Dietary Modulation Strategies

Fibers (lignocellulose) increase diversity and the abundance of butyrate producers (*Faecalibacterium, Roseburia*) (Hou et al., 2020), emphasizing NSP quality and quantity in low-protein diets (Hao et al., 2021; Ștef et al., 2009). Lysine restriction alters 12 genera and amino acid metabolism in pigs (Yin et al., 2018a); bacteria synthesize lysine from ammonium (Matthews, 2020), and lysine activates ERK1/2/NF-κB in the host (Han et al., 2018). Cross-feeding (lactate/acetate → butyrate) follows ecological principles (Horrocks et al., 2023). Ketogenic/vegan patterns tailor immune and metabolic health, mitigating dysbiosis caused by high meat and sweetener intake (Jadhav et al., 2023).

## 8 Next-Generation probiotics and live biotherapeutic products

While classical probiotics (e.g., *Lactobacillus* spp.) serve as general supportive supplements, next-generation probiotics (NGPs) and live biotherapeutic products (LBPs) represent a fundamental shift toward precision microbiome therapies targeting specific host-relevant microbial or metabolic signatures. Unlike dietary supplements, LBPs follow pharmaceutical-grade regulatory pathways and require rigorous proof of safety, quality, and efficacy for defined therapeutic claims (MTIG & Barberio, 2024).

Precision hinges on identifying robust microbial and metabolic signatures linked to health or production traits. Advanced computational approaches, such as non-negative matrix factorization (NMF), distill complex microbiome data into stable, predictive signatures that outperform those based on individual taxa (Duncan et al., 2025). Human studies show that machine learning models using merely five signatures rival those with 140+ species (Chen et al., 2025b; Duncan et al., 2025). Poultry exemplifies this through cecal signatures that co-enrich *Limosilactobacillus reuteri* and *Bacillus velezensis*, predicting high body weight and yielding efficacious consortia (Zhang et al., 2023a). This signature-to-consortium pipeline underpins NGP/LBP modalities (Table 2).

**Table 2:** Design framework for next-generation microbiome interventions in poultry.

Category	Target/Signature	Mechanism/Goal	Reference
Signature Consortia	L. *reuteri* + *B. velezensis* ↑BW	Synergistic growth restoration	(Zhang et al., 2023b)
GM Probiotics	Butyrate deficit/*Salmonella*	Targeted metabolite delivery (ARGFP)	(Amrofell et al., 2023)
Archaeal Modulators	H₂ methanogenesis→acetogenesis	↑FE, ↓CH₄ emissions	(Salahi & Abd El-Ghany, 2025; Yang et al., 2023)
Postbiotics	Low SCFAs/inflammation	Barrier/immunity modulation	(Ahmed et al., 2025; Salahi & Abd El-Ghany, 2024)
Synbiotics	Prebiotic ↑ probiotic colonization	Dual efficacy enhancement	(Yadegar et al., 2025)

**Abbreviations:** ARGFP: Antibiotic-Resistance-Gene-Free Plasmids; BW: Body Weight; CH₄: Methane; FE: Feed Efficiency; SCFA: Short-Chain Fatty Acids. GM; Genetically Modified.

NGPs/LBPs drive antibiotic-free (ABF) poultry production through dual action: sustaining growth while fortifying against dysbiosis caused by pathogens (*Salmonella, Clostridium perfringens, E. coli*) and nutritional stressors such as protein/lysine restriction (Jadhav et al., 2023). In line with this concept, non-antibiotic growth promoters such as sophorolipids have been shown to reshape the gut microbial composition toward a Firmicutes-dominant state and Lactobacillus enrichment, while suppressing inflammatory taxa, thereby supporting growth-promoting effects without antibiotic pressure (Huaiquipán et al., 2023). They enable precision resilience, extending antiviral protection via immune modulation against avian influenza and Newcastle disease (Ahmed et al., 2025).

Translational proof emerges from defined strains such as *Bacillus subtilis* 29,784, which shift microbiomes toward butyrate producers (Choi et al., 2021), and from fecal microbiota transplantation (FMT), which causally transfers traits such as improved gut morphology, reduced fat, and barrier function (Feng et al., 2024; Liu et al., 2024; Yan et al., 2021). FMT variability necessitates defined, signature-based LBPs (Fang et al., 2025). However, despite the growing predictive power of microbiome-based biomarkers and microbial signatures, most current evidence remains largely correlational. Future research should therefore progressively move from correlation to causation by establishing a chain of experimental evidence, including functionally validating core differential microorganisms through isolation, pure culture, and complementary experimental models. Advances in high-efficiency microbial cultivation and identification technologies will be essential for elucidating microbial functions, deciphering host–microbe interactions, and translating predictive microbial signatures into evidence-based next-generation probiotics and live biotherapeutic products (Chaudhari et al., 2021; Deng et al., 2025).

Archaeal modulation holds frontier potential. Despite low abundance (2–6 species), archaea play a pivotal role in regulating hydrogen metabolism (Salahi & Abd El-Ghany, 2025). Strategies shift from methanogenesis to acetogenesis, enhancing energy harvest and curbing methane emissions (Salahi & Abd El-Ghany, 2025; Yang et al., 2023). *Metatranscriptomics* confirms functional potency, as in hyperactive *Methanosphaera* spp. in pigs (Peng et al., 2022).

## 9 Microbial signatures as biomarkers

Microbial biomarkers are emerging as powerful, non-invasive tools for predicting growth performance, feed efficiency, and health outcomes in poultry, particularly within the framework of precision nutrition. However, their true predictive power resides not in a single microbial entity but in combining several indicators—taxonomic, genetic, and metabolic—into a multidimensional "microbial signature." Such multidimensional microbial signatures increasingly reflect ecosystem-level organization, in which metabolically interdependent networks and keystone taxa exert disproportionate control over gut function and stability (Steinert et al., 2025). These composite signatures, which reflect the functional state of the gut ecosystem, hold preliminary predictive potential as biomarkers (correlational; causal validation pending gnotobiotic/FMT). Rather than relying on individual taxa, predictive microbial signatures integrate multiple microbial features to improve classification performance and biological interpretability (Knights et al., 2011). Poultry FMT studies (e.g., expanded (Liu et al., 2024; Yan et al., 2021) transfer traits such as morphology and FCR, supporting causality for LP signatures. Objective evidence includes an algorithm combining five microbial markers that demonstrated high sensitivity (93.33%) and specificity (100%) in discriminating between high- and low-feed-efficiency broilers (Oliver et al., 2021).

Understanding these signatures provides crucial predictive insights into ecological and metabolic shifts within the host (Krummenauer et al., 2025). In poultry, concrete examples illustrate this concept. For instance, gut microbiome maturity strongly correlates with body weight and average daily gain in broiler chickens (Hu et al., 2025). Similarly, age-associated shifts in laying hens, such as enrichment of *Limosilactobacillus* and a decline in Prevotella, are closely linked to physiological states and egg production performance (Haque Shahid et al., 2026).

Translating this concept to the core challenge of sustainable diets—specifically low-protein, lysine-restricted formulations—requires a paradigm shift. A predictive microbial signature must move beyond a simple taxonomic census to capture the microbial community's functional capacity for nitrogen metabolism and recycling. Mouse studies show that low-protein diets (3%vs. 12% crude protein) activate microbial urea nitrogen salvage pathways, enabling gut bacteria to recycle host-derived urea into bioavailable nitrogen (Masuoka et al., 2020). In chickens, the ceca are the primary site of microbial nitrogen recycling, where urea is hydrolyzed and the resulting ammonia is reincorporated into microbial protein—a process critical for the host under protein-depleted conditions (Elling-Staats et al., 2022). Therefore, we hypothesize that a robust predictive signature for successful adaptation to low-protein conditions would feature: (1) elevated abundance and activity of ureolytic taxa (e.g., specific *Lactobacillaceae* and *Clostridiaceae* clades), (2) enrichment of ammonia assimilation genes (e.g., *gdhA, glnA*), and (3) a metabolic profile favoring beneficial SCFAs over nitrogenous waste (e.g., ammonia).

Supporting evidence spans species. A study of broilers fed a low-protein diet (–7% CP) found a time-dependent increase in cecal *Lactobacillaceae* that correlated with improved feed conversion during the finisher phase, suggesting it may be part of a "resilience signature" (De Cesare et al., 2019). Furthermore, foundational research in a pig model shows that lysine restriction (30%) significantly alters the gut microbiome, increasing diversity and enriching taxa such as Actinobacteria and Synergistetes, while also affecting host amino acid transporters and metabolism (Yin et al., 2018a). This provides crucial proof-of-concept that specific, measurable shifts in the microbial community are directly linked to host metabolic responses to amino acid restriction, a principle highly likely to translate to poultry.

Advancing toward comprehensive signatures requires integrating multi-omics data (16S rRNA with PICRUSt, shotgun metagenomics, and metabolomics), though metatranscriptomics for active pathways remains underrepresented. Combining metagenomics, metatranscriptomics, and metabolomics with machine learning provides a powerful framework for identifying diet-responsive microbial signatures (Naeem & Fatima, 2025; Oke et al., 2025) and developing predictive algorithms for precision nutrition, thereby enabling integration of microbial taxonomy, functional gene expression, and metabolite production (Wu et al., 2024). Recent poultry multi-omics studies further demonstrate that integrated microbiome–metabolome analyses can identify functional microbial–metabolic signatures associated with growth performance, supporting the application of multi-omics approaches for biomarker discovery and precision nutrition (Zhao et al., 2026). Metabolomics profiles, including short-chain fatty acids and nitrogenous compounds, serve as direct, functional readouts of microbiome activity and host nitrogen utilization efficiency (Oke et al., 2025). Despite promising correlative evidence linking microbial signatures to performance under low-protein and lysine-restricted diets, no standardized, validated signature is yet available for routine commercial poultry use. Bridging this translational gap requires: (1) identification of conserved LP/LR-responsive taxonomic, functional (e.g., gdhA/glnA-enriched), and metabolic signatures; (2) machine-learning integration to correlate these with zootechnical (FCR, weight gain) and sustainability metrics (nitrogen retention, ammonia emissions); (3) causal validation via gnotobiotic models and FMT; and (4) development of practical on-farm diagnostics (qPCR panels for signature taxa/genes, rapid SCFA assays). Achieving these steps will enable dynamic, signature-guided precision feeding that optimizes productivity, animal health, nitrogen efficiency, and environmental sustainability.

## 10 Conclusions

This scoping review confirms LPD/LR as master modulators of poultry gut microbial signatures—rewiring saccharolytic/SCFA pathways and mycobiota dynamics while suppressing proteolysis—thereby driving epigenetics, immunometabolism, and FCR via predictive biomarkers (optimized F/B ratios, ureolytic genes). Establishing validated, breed- and age-adjusted microbial signature thresholds will enable real-time, data-driven feed reformulation and personalized flock interventions. These signatures will guide NGPs and LBPs toward antibiotic-free production, improving nitrogen efficiency and reducing emissions. Nevertheless, the predominantly correlational evidence necessitates progression toward causal inference through longitudinal multi-omics studies, gnotobiotic and FMT validation, AI-driven personalization, and regulatory frameworks for LBPs. These advances will transform microbial signatures from descriptive patterns into actionable precision nutrition tools, elevating poultry welfare, profitability, and sustainability.

## Ethics statement

Not applicable. This study is a scoping review of previously published literature and did not involve human participants or animal experimentation.

## Declaration of generative AI and AI-assisted technologies in the manuscript preparation process

During the preparation of this work, the authors used generative AI tools for language refinement and grammar improvement, and for the creation of explanatory figures and graphical elements. After using these tools, the authors carefully reviewed and edited all content and take full responsibility for the accuracy, originality, and scientific integrity of the published article.

## Funding

This research did not receive any specific grant from funding agencies in the public, commercial, or not-for-profit sectors.

## CRediT authorship contribution statement

**Ahmad Salahi:** Conceptualization, Investigation, Methodology, Validation, Writing – original draft, Writing – review & editing. **Wafaa A. Abd El-Ghany:** Validation, Writing – review & editing, Visualization.

## Declaration of competing interest

The authors declare that they have no known competing financial interests or personal relationships that could have appeared to influence the work reported in this paper.

## Data Availability

No new sequencing data, original code, or primary experimental datasets were generated in this PRISMA-ScR scoping review. All data supporting the findings of this synthesis were derived from previously published studies and are fully cited within the reference list. No custom code or software was developed that is essential for the conclusions of this article.
